# Case Report: Anomalous Experience in a Dissociative Identity and Borderline Personality Disorder

**DOI:** 10.3389/fpsyt.2022.662290

**Published:** 2022-07-18

**Authors:** Hugo André de Lima Martins, Valdenilson Ribeiro Ribas, Ketlin Helenise dos Santos Ribas, Luciano da Fonseca Lins, Alessandra Ghinato Mainieri

**Affiliations:** ^1^Unidade do Cérebro, Surubim, Brazil; ^2^Instituto do Cérebro de Pernambuco, Jaboatão dos Guararapes, Brazil; ^3^Universidade de Pernambuco (UPE), Garanhuns, Brazil; ^4^Federal University of Rio Grande do Sul, Porto Alegre, Brazil

**Keywords:** borderline, personality, dissociation, mediumship, anomalous experience, case report

## Abstract

**Introduction:**

Dissociative identity disorder, formerly called multiple personality disorder, is a rupture of identity characterized by the presence of two or more distinct personality states, described in some cultures as an experience of possession.

**Objective:**

The case of a 30-year-old woman with dissociative identity disorder and borderline personality disorder associated with a previous history of anomalous experience was reported.

**Case Report:**

A 30-year-old woman who fulfilled the DSM-5 criteria for dissociative identity disorder and borderline personality disorder reported the presence of unusual sensory experiences (clairvoyance, premonitory dreams, clairaudience) since she was 5 years old. The patient told that for 12 months she presented episodes in which a “second self” took charge of her actions: she would then speak with a male voice, become aggressive, and require several people to contain her desire for destruction. After 3 months of religious follow-up, and accepting her unusual experiences and trance possessions as normal and natural, she had significant improvement.

**Conclusion:**

When approaching DID and BPD patients, it is necessary to observe the anomalous phenomena (in the light of) closer to their cultural and religious contexts, to promote better results in the treatment of their disorders, which has not been explored in the treatment guide.

## Introduction

Pierre Janet was the first to describe, in 1889, dissociation as disaggregation of the unity of experience at the mental level (1). Dissociative identity disorder (DID) is characterized by two or more distinct identities or dissociated personalities.

Dissociation is characterized by a disturbance in integrated dimensions of the mind such as consciousness, attention, memory, and perception of the environment. This dispersion of the sense of self-oneness causes deterioration of chronological, biographical, and perceptive unity (1–4).

Dissociative disorders seem to arise because of the transaction between genetic factors, which determine an individual's biological vulnerability and environmental condition (5). Among the latter, the socio-cognitive model highlights the importance of social-cultural self-oneness as the cause of deterioration of chronological, biographical, and perceptive unity (1, 2, 4), while the trauma model, which has received more support and was studied more, underlies the role of traumatic life experiences (6, 7). Very recently, aligned with the transdiagnostic model, it was proposed that dissociation can be understood as failures of normally adaptive systems and functions (8).

Some theorists believe that the alleged personalities would be an attempt to defend the weakened ego from childhood trauma or abuse that occurs in more than 80% of cases of DID (1, 4). In such cases, dissociation acts as a self-hypnotic defense mechanism that provides conditions for the individual to cope with trauma (1, 9–11). Other researchers, more skeptical, think that DID is not a real condition, but a disorder produced by doctors or cultural influence in highly hypnotizable and “suggestible” patients (12). These are the two main lines of thought about the etiology of DID, although the latter seems to have less empirical support.

Symptoms of dissociation are present in a variety of mental disorders such as DID and post-traumatic stress disorder (PTSD) (13). Borderline personality disorder (BPD) is a very serious psychiatric condition characterized by severe affective instability and impulsivity, associated with problems in self-image and interpersonal relationships (1). Transient, stress-related severe dissociative symptoms” serve as a criterion for borderline personality disorder (14). Most patients with BPD present episodes of identity confusion, derealization, depersonalization, and dissociative amnesia (4).

‘Anomalous Experiences' (AE) is a term proposed to designate unusual experiences which are considered ‘outside the ordinary explanations' (hallucinations, synesthesia, and experiences interpreted as telepathic, paranormal, among others), without assuming psychopathological implications. These phenomena are reported in all cultures and in all times of humanity, which were the object of study of official science in the late nineteenth and early twentieth centuries, but which only in recent years have returned as interesting areas in the academic field. Some examples of AE are clairvoyance, premonitions, xenoglossia, and mediumistic incorporations (15–17). In general, authors make a distinction between those who present AE, which represents a form of non-pathological dissociation, from those who fulfill criteria for DID which causes discomfort and suffering (18).

We report a case of DID associated with BPD that draws attention to the presence of AE, such as clairvoyance, premonitory dreams, and clairaudience, from 5 years of age, preceding the onset of possession-type dissociative identity crises in more than two decades. The patient gave informed consent for this case report and the study was approved by the ethical committee with number 3,605,351. There was no funding for this research.

## Clinical Case

A 30-year-old woman presented with a history of repeated episodes of identity disturbance characterized by a marked change in behavior, aggression, psychomotor agitation, and voice change (from female to male voice). The episodes started in March 2018 and lasted from 10 min to 6 h, at an average frequency of 3 times a week.

The patient generally had a partial or total recollection of events. She was ashamed of the people who witnessed the episodes because she felt ridiculed. She could not avoid possession, which happened in places like churches, at the school where she worked as a teacher, at home, and at the doctor's office. Since the beginning of the condition, she had been showing moderate social isolation, because the community where she lives believed that she was possessed by an evil entity. She had several days of absenteeism at work due to crises and failed several medical treatments.

She often attended masses of the Catholic Church, where these occurrences were not well regarded. After one almost uncontrollable crisis, the patient broke the pews of the church during the service. The priest decided to submit her to a ritual of exorcism, which consisted of prayers, holy water, and crucifix presentation. During the session, the patient attacked eight people, including the priest, who had his clothes torn.

The treatment was abandoned, as the patient's family was embarrassed by the amount of stuff broken and people injured during the “possession” state, besides not obtaining satisfactory results, which diminished the interest in continuing this procedure.

She did not intend any kind of secondary gain with that disorder and, most of the time, she would get physically exhausted along with the trance: at the start, she would get overly strong and, up to the end, she would be very weak. Practitioners of her religion (Catholic) were not used to dealing with these manifestations. As time went by, the trance episodes increased in frequency and intensity and she felt more isolated at work and in her social relationships. Concomitantly with those religious sessions, the patient went through several unsuccessful psychiatric treatments over a year. Our service was then referred to the patient by another colleague.

At the initial consultation, she was quite frightened, as she had several embarrassing situations and was profoundly affected by the fact that she had no control over her body. Personal background: She denies a history of abuse or neglect. She said that at the age of 5, she was in the recovery room of a tonsillectomy surgery when she had a vision of a spiritual entity, dressed in light clothes, who told her about the importance of ethical and moral behavior in life.

She reported the vision to her family, but it was not taken seriously, and they mocked her. The most striking case was that of a repetitive dream with an unknown middle-aged man, whom she met after a few months at a horse farm.

She had other similar dreams between the ages of 5 and 11. When she was 10 years old, she was awakened in the middle of the night by an entity who stated to be her grandfather, who had died 2 years before. She wrote a letter dictated by her deceased grandfather to her father regarding personal matters that were completely unknown to her. The signature showed some resemblance to her grandfather's. The next day, her parents read the letter and said they were sure it was the devil's work and tore the entire manuscript. In her early teens, she had the feeling that a spiritual entity intended to have sex against her will. She was very bothered by the feeling that someone was running his hands all over her body, including her private parts. She did not talk to others about these feelings, because she was afraid someone might think she was “crazy.” After starting her sexual life, she had an invisible and unusual sensory experience as rape-like provoked by supposed bad spiritual beings. These sensations were so threatening that they led her to frequent suicidal thoughts. The patient reports that the episodes were completely unwanted and aroused a feeling of despair, with an intense resemblance to reality.

She made several suicide attempts through lethal methods such as hanging, drowning, moving motorcar, and electric shock, always being driven by a male voice that guided her. At several moments, she completely lost her mind and body control during the episodes and assumed that an external entity commanded her. The patient consulted several specialists, who gave various diagnoses such as depression, anxiety disorder, schizophrenia, and panic disorder. She took nortriptyline 75 mg daily for 6 months, fluoxetine 40 mg daily for 4 months, escitalopram 20 mg daily for 3 months, risperidone 6 mg daily for 4 months, quetiapine 600 mg at night for 4 months, some of them in combination in the last year, with no clinical improvement. She did not have any therapeutic benefits from these drugs, although she experienced all the side effects. In this case, there was probably a good adherence to pharmacological treatment, although it is not been proven by measuring the plasma level of the substances.

Among the symptoms, the patient said that she heard voices, saw figures, often dreamed of deceased people, thought randomly about things that came to happen after a while, believed to write automatically and unconsciously, driven by a force external to her thinking. She complained of many very rapid mood swings, fear of abandonment, and an intense feeling of emptiness.

The patient was born by transpelvian delivery and had normal neuropsychomotor development. She had a tonsillectomy at 5 years of age. She never attended psychological counseling. The patient denied a history of childhood abuse or neglect. The patient spent her childhood and adolescence in a situation of low socioeconomic level. Her mother had behavioral problems but never went through any kind of treatment. There is no information on family health and AE history.

In the mental state examination, the patient did not present alterations except for a very anxious mood. The structure of thought was completely normal. The physical and neurological examination revealed no abnormalities. The patient obtained 45 points in the Beck anxiety inventory (BAI) and 45 on the dissociative experiences scale (DES). Lab Tests, Brain MRI, and 3 repetitive EEGs were normal.

The patient fulfilled all criteria for diagnosis of dissociative identity disorder according to DSM-V: characterized by two or more distinct personality states (also called alter egos or self-states or identities). There is also an inability to recall daily events, important personal information, and/or traumatic or stressful events, all of which typically would not normally be lost with normal forgetting. The symptoms caused social and professional harm, were not part of a context accepted by religious practice, and were not due to a physiological effect of substances or other medical conditions.

The patient refused psychotherapy for economic reasons and decided to attend Spiritism, which accepts communication among the living and the dead as part of its doctrinal framework. Spiritism started initially in France as a spiritualistic movement developed in the 19th century, and nowadays it has spread around the world. In Brazil, it is the third-largest religion and its practices strongly emphasize controlled psychotic and dissociative experiences called mediumship. Mediumistic practices are not reimbursed but are considered charitable voluntary work (19, 20).

After 3 months in the new religious order, where her dissociative manifestations were naturally accepted, without the interpretation that it would be the result of the influence of the supreme evil, the patient had marked improvement in anxiety symptoms, reducing the BAI score to 26, becoming able to speak spontaneously about her crises and very rarely presented the picture outside the appropriate religious context. People sometimes refer to fear in participating in Spiritism meetings due to the lack of proper information about the safety of the procedure. The patient denied any concern about it, although she kept discretion about her treatment for people outside its context, due to the fear of suffering prejudice. She also returned to her social and occupational activities.

## Discussion

The patient reported dreams that seemed very real to her. Some authors have correlated dissociative symptoms with sleep disorders (21), even highlighting the role of the latter as a cause of dissociation (22–25). For instance, sleep improvement reduces dissociative symptoms (26). When sleep and dream systems are impaired, the memory process during (REM) sleep becomes unregulated and it may as well induce dissociative symptoms (24).

The reported case shows a patient with unusual and premonitory dreams in her childhood, which seem to be related to a current psychopathological condition. A very interesting study evaluated the frequency of dream recall and the experience of unusual dreams, longitudinally, in children of both genders, aged between 10 and 11 years, for 2 years, with an initial assessment and after 12 and 24 months. The tendency to have unusual dreams, such as repetitive dreams, remembering dreams over a long period, or dreams that cannot be understood, was associated with internalizing and externalizing behavioral problems reported by the adolescents' parents (27).

A particular type of dreaming is designated by the term lucid dreaming in which the dreamer is aware of dreaming (28, 29). Furthermore, in this condition, control (the capacity to change the dream events) and dissociation represent the other criteria (30). In this report, the patient had no control over the dream plot and perceived it as real and unpleasant. Lucidity in dreaming has been linked with positive rather than negative emotions (24), but when the person has no control over the dream, which seems to be more common, lucid dreaming is associated with psychopathological distress and several types of symptoms (31).

There were not remarkable events that might be considered very traumatic in the patient's childhood and adolescence. The possibility of abuse and neglect was extensively researched and no evidence was found. It has been widely documented in specialized literature that in most cases of DID there is a serious and traumatic event during the patient's life, which might justify the onset of dissociative symptoms (12, 32, 33). Otherwise, minor traumas, such as surgery at age 5 with hallucination, associated with an invalidating attitude from the family who mocked her, could lead to the DID.

The patient heard commanding voices ordering her to commit suicide, which resulted in several attempts. Auditory hallucinations present in epileptic seizures are generally elementary, characterized by repetitive and simple sounds (4). She also fulfilled the criteria for borderline personality disorder as an unstable sense of self; chronic feeling of emptiness; inappropriate and intense anger; history of recurrent suicidal behavior; and severe dissociative symptoms, which justify the absence of effectiveness in pharmacological treatment.

Recently, some researchers have studied the accuracy of the information contained in a letter supposedly dictated by a deceased person to the influential Brazilian “medium” Chico Xavier, encountering highly specific hits (34). The patient's letter was torn and could not be evaluated. Otherwise, the automatic writing may be explained by dissociative absorption and imaginative involvement, which is not necessarily pathological and is characterized by a tendency to become immersed in a stimulus while neglecting to attend to one's surroundings (35). As a quite common dissociative process, automatic writing may be characterized by a diminished sense of agency (36) and it can alternatively explain why someone assigns the authorship of the letter to someone else.

Since childhood, the patient had AE, such as clairvoyance and premonitions, which were never studied with attention, probably because this is an unknown field to the lay public and poorly explored by the scientific community. Although AE, in general, occurs in people with no mental disorders, the present case pointed to the possibility of overlapped events like AE and psychopathologic alterations as the patient underwent various suicide attempts during her life and it is in general associated with a mental disorder. There has been recently a new tendency to make AE an object of study in the natural sciences again (16, 37).

The patient was refractory to various drug therapy regimens, which are widely cited in the literature related to the dissociative identity disorder (10). Unfortunately, there was no opportunity to perform psychotherapy to integrate identities, because the patient lacked the financial resources to do so.

A very interesting fact was the clinical improvement of the patient, evidenced by both the mental state examination and the use of a psychometric instrument (BAI) after being welcomed into a religious community (Spiritism) that accepted possession as part of its doctrinal structure. According to the DSM-5, one of the criteria for diagnosing DID is that it does not belong to a widely accepted religious or cultural practice (38).

In low- and middle-income countries, psychotic experiences are present at least occasionally in more than 90% of the people. There is an assumption that these experiences are more culturally accepted in these countries, which justifies the numbers. For these individuals, lower distress is predicted by spiritual appraisals and better social support from family and friends (39). Possibly, this approach influenced the favorable outcome in this case report.

## Conclusion

This study explored the importance of cultural and religious contexts, and consequently, their interference in the evolution of patients with anomalous experiences and dissociative disorder, and explored the relationship between anomalous experience and dissociative disorder, expanding the explanatory possibilities for this disorder.

The main strength of this report is showing an alternative way to manage the complex DID. The limitation is related to the type of study (case report) and to the possibility that the patient improved by accepting her AE as natural, which could, in theory, happen in supportive psychotherapy.

Although it should be acknowledged that parts of some cases of DID have traumatic etiological factors, the present case reflects the positive association between the event and the trajectory of the dissociation that changed once she found a social or religious group that accepted her possession crises as a natural event, providing a positive framework not just for the present symptoms, but also a possible explanation to the different events that she had all along her life.

## Data Availability Statement

The raw data supporting the conclusions of this article will be made available by the authors, without undue reservation.

## Ethics Statement

The studies involving human participants were reviewed and approved by Federal University of Pernambuco. The patients/participants provided their written informed consent to participate in this study. Written informed consent was obtained from the individual for the publication of any potentially identifiable images or data included in this article.

## Author Contributions

HM contributed substantially to the design of the study: he was responsible for the acquisition of data (articles) for the work and for the critical review of the work and also participated in the writing of the introduction, in the critical review of the case report, and in preparing the abstract. VR was responsible for the choices of scientific articles related to the study, participated with substantial contributions to the design of the study, and in discussions about interpretations of the chosen articles and also as a corresponding author. KR contributed to the writing of the case report, participated in discussions on the choices and interpretations of the chosen scientific articles and submission to the ethics and research committee. LL participated in the discussions and interpretations of the scientific articles related to the study and in the critical review. AM participated in all discussions related to data and the study phenomenon, agreed to be responsible for all aspects of the work, and ensuring that issues related to the accuracy or integrity of any part of the work are investigated. All authors contributed to the article and approved the submitted version.

## Conflict of Interest

The authors declare that the research was conducted in the absence of any commercial or financial relationships that could be construed as a potential conflict of interest.

## Publisher's Note

All claims expressed in this article are solely those of the authors and do not necessarily represent those of their affiliated organizations, or those of the publisher, the editors and the reviewers. Any product that may be evaluated in this article, or claim that may be made by its manufacturer, is not guaranteed or endorsed by the publisher.
